# Evaluation of electrocardiographic parameters in amputee football players

**DOI:** 10.3389/fpsyg.2023.1189712

**Published:** 2023-07-24

**Authors:** Ahmet Kurtoğlu, Ertuğrul Kurtoğlu, Alkame Akgümüş, Bekir Çar, Özgür Eken, Ioan Sârbu, Carmen Iulia Ciongradi, Dan Iulian Alexe, Iuliana Laura Candussi

**Affiliations:** ^1^Department of Coaching Education, Faculty of Sport Science, Bandirma Onyedi Eylul University, Balikesir, Türkiye; ^2^Department of Cardiology, Medical Faculty, Malatya Turgut Ozal University, Malatya, Türkiye; ^3^Department of Cardiology, Medical Faculty, Bandirma Onyedi Eylul University, Balikesir, Türkiye; ^4^Department of Physical Education and Sport Teaching, Faculty of Sport Sciences, Bandirma Onyedi Eylul University, Balikesir, Türkiye; ^5^Department of Physical Education and Sport Teaching, Faculty of Sport Sciences, Inonu University, Malatya, Türkiye; ^6^Second Department of Surgery—Pediatric Surgery and Orthopedics, “Grigore T. Popa” University of Medicine and Pharmacy Iasi, Iași, Romania; ^7^Department of Physical and Occupational Therapy, Faculty of Movement, Sports and Health, Sciences, “Vasile Alecsandri” University of Bacau, Bacau, Romania; ^8^Clinical Surgery Department, Faculty of Medicine and Pharmacy, “Dunărea de Jos” University, Galați, Romania

**Keywords:** amputation, cardiac function, disability, electrocardiography, football

## Abstract

**Objective:**

The present study aimed to compare electrocardiographic (ECG) parameters of amputee football players (AF) with football players without disability (FP) and sedentary individuals without disability (SI).

**Methods:**

A total of 32 participants (AF = 9, FP = 11, SI = 12) were included in the study. ECG parameters including P-wave amplitude, P-wave duration, PR interval, QRS duration, RR interval, QT interval, corrected-QT interval (QTc), ST segment duration, Tp-e duration, Tp-e/QT and Tp-e/QTc ratios were assessed in all the study participants by using a 12-lead ECG device. OneWay ANOVA Test was used for statistical analysis.

**Results:**

Of all ECG parameters, P-wave amplitude and QTc were significantly higher in the AF group in comparison to FP and SI groups. QRS duration was found to be lower in the AF group when compared to FP and SI groups. Myocardial repolarization parameters including Tp-e duration, Tp-e/QT and Tp-e/QTc ratios were similar between groups, as were other parameters such as P-wave duration, PR interval, RR interval, QRS duration and QT interval.

**Conclusion:**

It was found that some ECG parameters of amputee football players differ from those with non-disabled players and non-disabled sedentary individuals. These different parameters were within normal limits.

## 1. Introduction

Amputee football (AF) is classified as a high-interval sport with periods of high intensity (Maehana et al., 2018; Nowak, 2020). Many features such as power, speed, endurance, balance, agility and endurance should be at a high level in AF players (Ozkan et al., 2012). Short-term high-intensity power generation and aerobic capacity play an important role in the performance of AF players (Yazicioglu et al., 2007). This is an indication that AF players will not expend more energy during the game compared to their healthy peers (Tatar et al., 2018).

Previous studies showed that traumatic above-knee amputations are associated with increased long-term cardiovascular morbidity or mortality (Bakalim, 1969; Van Alsté et al., 1985; Modan et al., 1998; Chin et al., 2006; Petrini et al., 2019). In the study, in which war veterans were examined for a long time in a controlled manner after World War II, it was found that individuals with unilateral amputation had cardiac problems 1.5 times and individuals with bilateral amputations had cardiac problems 3.5 times (Modan et al., 1998). In another study, individuals with above-knee amputation after the Vietnam War were compared with individuals with elbow amputations and it was determined that individuals with above-knee amputation showed more hypertensive characteristics (Rose et al., 1987). In this context, there are studies comparing the cardiac structures of amputee individuals with other amputees, as well as with healthy individuals (Vollmar et al., 1989; Li et al., 2022).

It is well known that the heart undergoes a number of structural and functional adaptations after strenuous and repetitive exercise (Paterick et al., 2014). Compared to sedentary individuals with an athlete’s heart, athletes generally have some morphological improvements in left ventricular systolic and diastolic parameters and in the left atrium (Lee et al., 2022). Amputee athlete’s heart responds differently to exercise compared to healthy athletes (Kurtoğlu et al., 2023). For this reason, it is thought that analyzing the cardiac functions of amputees with different methods may play an important role in the selection of exercises to be applied to these individuals.

Electrocardiography (ECG) is an important application in the early diagnosis of cardiac diseases. It is a reliable prognostic test for identifying possible cardiac problems in athletes, especially before starting sports (Hachamovitch et al., 1998). ECG is a method that is easy to apply and provides important findings about the cardiac data of the athlete (Marwick et al., 2003). In ECG recording, the electrical potential created by the current in the heart is recorded by electrodes placed on the skin (Walinjkar and Woods, 2017). In routine ECG, P-wave amplitude, P-wave duration, PR interval, QRS duration, QT interval and ST segment are evaluated (Tan et al., 2009). In recent years, some other ECG parameters such as Tp-e interval, Tp-e/QT and Tp-e/QTc ratios were found to be related in arrhythmogenesis (Kors et al., 2008). These parameters together with QT and QTc intervals have been accepted as electrocardiographic markers to predict ventricular arrhythmias and cardiovascular mortality (Castro Hevia et al., 2006).

Considered in this context, risk factors for this amplified morbidity and the involved pathophysiologic mechanisms have not been comprehensively studied. The number of studies evaluating the optimum intensity of physical exercise in amputees and their possible long-term consequences is also limited. Previous studies consistently showed that some cardiovascular structural abnormalities may occur over time due to strenuous activities in football players without disability (Egger et al., 2022). This abnormalities may gradually become more pronounced in disabled football players due to higher energy consumption during activities and some changes in cardiovascular system due to amputation. Although these adaptations in disabled players does not have to reach overt structural cardiovascular changes, it may be hypothesized that electrocardiography may change in this population. In addition, it is intriguing to analyze a broad of depolarization and repolarization parameters electrocardiographically in AF players. Furthermore, there is not much data regarding ECG findings in this population. Therefore, we aimed to assess a broad range of ECG parameters in AF players.

## 2. Materials and methods

### 2.1. Participants

The minimum sample size for the present study was calculated using G-power software 3.1.9.7. (University of Dusseldorf, Dusseldorf, Germany; Franz et al., 2007). The present study utilized *a priori* and F tests to estimate statistical power based on the study’s design, including ANOVA with fixed effects and omnibus one-way testing. The significance level (α err prob) was set at 0.05, with a minimum effect size of 0.60 and three groups. A power analysis was conducted, yielding a minimum sample size of 30 participants to achieve a true power of 80.0% for statistical significance. The study population composed of those who playing in elite amputee football league for at least 1 year (AF group), individuals without disability who playing football actively in at least amateur league teams (HF group), and sedentary individuals without disability who perform less than 2 h of weekly exercise (SI group). The weekly training frequency of the AF group was 3.33 ± 0.50 days, the training time in a particular day was 91.66 ± 10.30 min, and the sports age was 9.66 ± 4.30 year. The AF group consisted of Brazil (2 people), Iran (3 people), Morocco (1 person) and Turkey (3 people) national team players. Individuals who had hypertension, diabetes, coronary artery disease, cardiac arrhythmia, thyroid disorders, moderate to severe heart valve disease, active infection, smoking and using performance enhancing products and alcohol were excluded from the study. Because females do not participate in amputee football league, the study population inherently composed of male participants. Initially, 37 volunteers were recruited, of whom 1 volunteer were excluded from moderate valvular heart disease, 1 from frequent premature ventricular contraction (a cardiac arrhythmia), 1 from active urinary infection, 2 from smoking. A total of 32 study participants were eventually included in the study, of which 9 participants were in the AF group, 11 participants in the SF group, and 12 participants in the SI group. The AF group had 2 arm amputations, 3 transtibial amputations, 2 transfemoral amputations, and 2 hip disarticulations. All of the AF participants were trauma-related amputations.

All ECG measurements of the participants in the study were done by a cardiologist. Participants were warned not to drink food or drink other than water at least 2 h ago before the measurement. In order for the ECG results to be affected by the circadian rhythm (Nakagawa et al., 1998), the participants were awakened no later than 08:00 in the morning after going to sleep no later than 00:00 the night before the test. ECG was taken in a quiet room in the hospital at 15:30 on different days.

BMI calculation of disabled population differs from nondisabled population. For this reason, BMIs of amputees were corrected according to the Amputee Coalition (AC). Based on the published sources and expert opinion, AC calculator corrects for proportions of total BM missing based on the following percentages: foot (Symes) = 1.30%, transtibial = 3.26%, transfemoral = 9.96%, and hip disarticulation/hemipelvectomy = 11.83%. This correction does not differ for men or women, unlike other estimates which are slightly higher for women: transtibial = 3.355% and transfemoral = 10.712% (Osterkamp, 1995; Durkin and Dowling, 2003; Frost et al., 2017). Estimated BM was calculated as:


$$\begin{array}{l} \mathrm{Estimated}\;\mathrm{BM}=\left(\mathrm{BM}\;\mathrm{without prosthesis}\right)/\\ \;\;\;\;\;\;\;\;\;\;\;\;\;\;\;\;\;\;\;\;\;\;\;\;\;\;\;\;\;\;\;\;\;\;\;\;\;\;\;\;\left(1\mathrm{.0}-\mathrm{AC}\%\mathrm{converted to decimal fraction}\right)\; \end{array}$$

### 2.2. Assessment of ECG

After collecting participants’ demographic data, ECG recordings were done in the supine position using a 12-lead ECG machine (Nihon Kohden Cardiofax, Japan) in a silent room. Measurements were performed at a rate of 50 mm/s and an amplitude of 20 mm/mV to increase sensitivity of measurements. ECG recordings were recorded on paper with a spacing of 20 ms between two vertical lines and 0.5 mm between two horizontal lines. Accordingly, P-wave amplitude, P-wave duration, PR interval, QRS duration, RR intervals, QT interval, QTc interval, ST segment, Tp-e duration, Tp-e/QT, and Tp-e/QTc ratios were calculated. Heart rate was also calculated at the same time. P-wave duration was measured from the beginning of the P-wave to the ending of P-wave. P-wave amplitude was calculated from the baseline P wave to the peak of the P wave. The PR-interval was measured from the beginning of the P-wave to the beginning of the following QRS complex. QRS duration was measured from the first positive or negative deflection of QRS wave to the end of QRS wave where it unites baseline. The QT interval was measured from the beginning of the QRS complex to the end of the T wave. The QTc duration was calculated by measuring the time from the onset of the QRS connection to the end of the T wave and dividing by the square root of the RR interval using the Bazzet formula (Desai et al., 2003). The Tp-e interval was defined as the difference between the peak of the T wave and the end of the T wave. All the measurements were then divided by 2 because of two-fold increase in paper recording rate and amplitude. In case of ratio measurements such as QTc, half-fold update in individual parameter was done before entering parameters into any formula. An electronic caliper was used to minimize measurement errors. All evaluations were evaluated by a specialized cardiologist. Participants who met the inclusion and exclusion criteria were warned not to exercise before the study. Participants were advised not to consume any food or drink other than water for at least 3 h before the measurements. The coefficient of variation between observers and within observations (calculated by dividing the standard deviation of two observations by their mean and expressed as a percentage (%) was less than 5%).

### 2.3. Ethical consent

Participants were informed of the purpose and importance of the study. Participants were informed of the possibility of leaving the study at any stage. Accordingly, the voluntary consent form was signed by the each participant. The study was conducted in accordance with the principles of the Declaration of Helsinki. The necessary permissions to conduct the study were obtained from the football clubs. The necessary permissions were obtained from the Ethics Committee for Non-Interventional Research of Bandırma Onyedi Eylul University (ethical number 2022-154).

### 2.4. Statistical analysis

The data in the research were made with SPSS (Version 28, IBM, United States) package program. The normality analyzes of the data were done using Shapiro–Wilk test because the number of participants was less than 30 (Mohd Razali and Bee Wah, 2011). Normality testing of the data revealed that the distribution was within normal limits. Accordingly, descriptive statistics were presented as the mean (x̄) and standard deviation (ss). To determine whether there were statistically significant differences between the groups, a parametric test known as One-Way ANOVA was utilized. In addition, Bonferoni *post-hoc* test was performed for comparisons between groups. In addition, the effect size was determined according to the Partial Eta Square (η^2^p) formula. In pairwise comparisons, the effect size was determined using Cohen’s d formula. In this context, the effect size yielding 0.2 was accepted as small, 0.5 as medium, and 0.8 as large (Cohen, 1988). The significance level of the study was set at *p* < 0.05.

## 3. Results

A total of 32 participants were included in the present study. There was 9 participants in the AF group, 11 participants in the HF group and 12 participants in the SI group. All the participants were male. The groups were similar regarding age, weight, height and BMI. Baseline characteristics of the study population are given in Table 1.

**Table 1:** Baseline characteristics of the study population.

Parameters	AF (*n* = 9)	FP (*n* = 11)	SI (*n* = 12)	*p*
Age (years)	25.55 ± 4.85	23.90 ± 3.38	24.50 ± 5.24	0.725
Weight (kg)	75.88 ± 14.42	71.90 ± 9.47	73.00 ± 12.86	0.274
Height (cm)	174.44 ± 10.80	178.09 ± 6.84	179.66 ± 3.47	0.765
BMI (kg/m^2^)	24.68 ± 2.49	20.50 ± 7.09	22.64 ± 4.08	0.199

^*^p < 0.05; AF, amputee football players; FP, healthy football players; SI, sedentary individuals.

**Figure 1:**
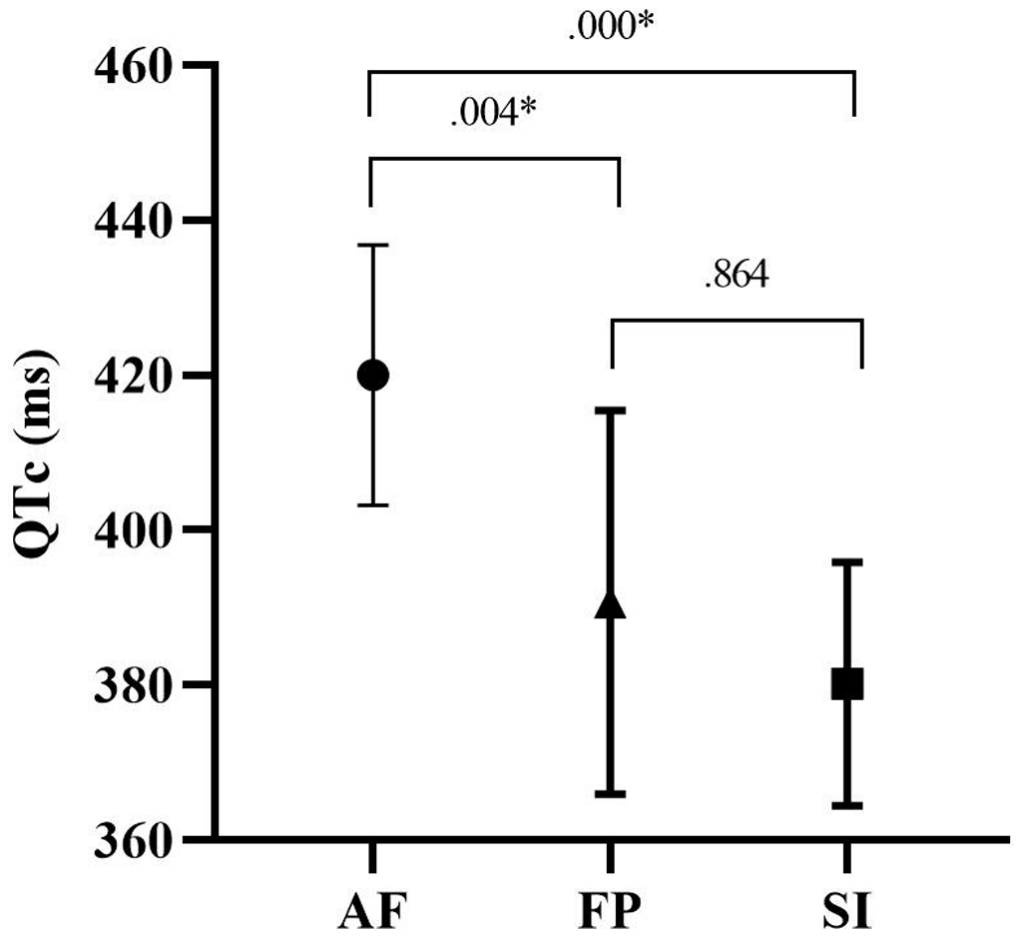
QTc durations of the study groups.

**Figure 2:**
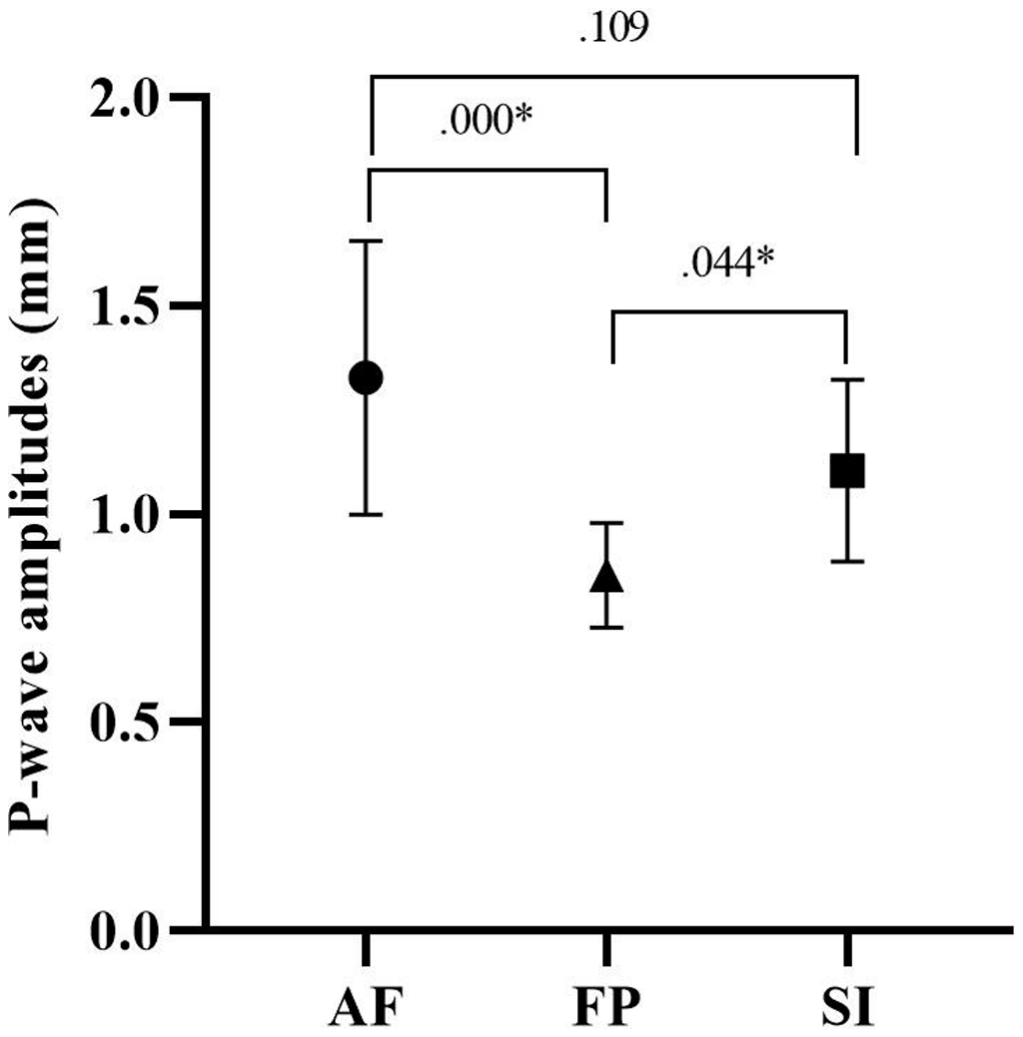
P-wave amplitudes of the study groups.

Regarding basic ECG parameters, heart rate, P-wave duration, PR interval and QT interval were similar between the study groups (Table 2). Myocardial repolarization parameters, which are Tp-e duration, Tp-e/QT and Tp-e/QTc ratios, were also not different between the study groups. The QRS duration was found to be lower in the AF group when compared to SI group but not to HF group, although it was statistically borderline (*F* = 3.407, *p* = 0.047, η2p = 0.190). The QTc value in the AF group (420.00 ± 16.80 ms) was significantly higher than in HF group (388.90 ± 24.22 ms) and SI (380.16 ± 15.74 ms; *p* = 0.004, *p* = 0.000, respectively; Figure 1; Table 2). P-wave amplitude were statistically significantly different between groups. *Post-hoc* analysis showed that P-wave amplitude was significantly higher in the AF group (1.32 ± 0.32 mm) in comparison to HF group (0.85 ± 0.12 mm) and but not to SI group (1.10 ± 0.21 mm; *p* < 0.001 and *p* = 0.109, respectively; Figure 2; Table 2).

**Table 2:** ECG parameters of the study population.

Parameters	Groups	X̄ ± Sd	F	ES	*p*
HR (beats)	AF	81.66 ± 13.36	1.364	0.99	0.272
	FP	76.45 ± 12.46			
	SI	73.08 ± 9.78			
QTc	AF	420.00 ± 16.80	11.570	3.78	0.001^*^
	FP	388.90 ± 24.22			
	SI	380.16 ± 15.74			
P-wave amplitude (mm)	AF	1.32 ± 0.32	10.528	0.39	0.000^*^
	FP	0.85 ± 0.12			
	SI	1.10 ± 0.21			
P wave duration (ms)	AF	92.44 ± 11.12	0.904	0.74	0.416
	FP	92.09 ± 8.40			
	SI	97.08 ± 10.10			
PR-interval (ms)	AF	153.33 ± 16.39	1.441	1.25	0.253
	FP	140.36 ± 14.06			
	SI	147.50 ± 19.94			
QRS (ms)	AF	77.22 ± 6.18	3.407	1.53	0.047^*^
	FP	84.63 ± 5.60			
	SI	88.33 ± 13.87			
QT-interval (ms)	AF	363.33 ± 35.35	1.013	1.36	0.375
	FP	347.27 ± 30.68			
	SI	346.25 ± 23.65			
Tp-e (ms)	AF	100.00 ± 19.68	2.372	1.48	0.111
	FP	91.72 ± 10.58			
	SI	85.83 ± 13.78			
R-R interval (ms)	AF	741.11 ± 108.56	1.646	3.67	0.210
	FP	813.18 ± 142.97			
	SI	843.33 ± 130.19			
Tp-e/QTc	AF	0.23 ± 0.04	0.388	0.026	0.682
	FP	0.23 ± 0.03			
	SI	0.22 ± 0.03			
Tp-e/QT	AF	0.27 ± 0.04	1.875	0.07	0.171
	FP	0.26 ± 0.02			
	SI	0.24 ± 0.02			

HR, Heart Rate; QTc, Corrected QT; Tp-e, Tpeak-end; ^*^p < 0.05; AF, amputee football players; FP, healthy football players; SI, sedentary individuals.

Figure 1 shows the *post-hoc* test results of participants’ QTc values. According to this, the mean QTc value of the AF group (420.00 ± 16.80 ms) was significantly higher than the mean value of the HF group (388.90 ± 24.22 ms) and SI (380.16 ± 15.74 ms; *p* = 0.004, *p* = 0.000).

Figure 2 shows the *post-hoc* test results of participants’ p-wave-lengths. According to this, although the p-wave length of group AF (1.32 ± 0.32 mm) was higher than that of groups HF (0.85 ± 0.12 mm) and SI (1.10 ± 0.21 mm), the difference between AF and HF (*p* = 0.000) was higher than the difference between HF and SI (*p* = 0.044).

**Figure 3:**
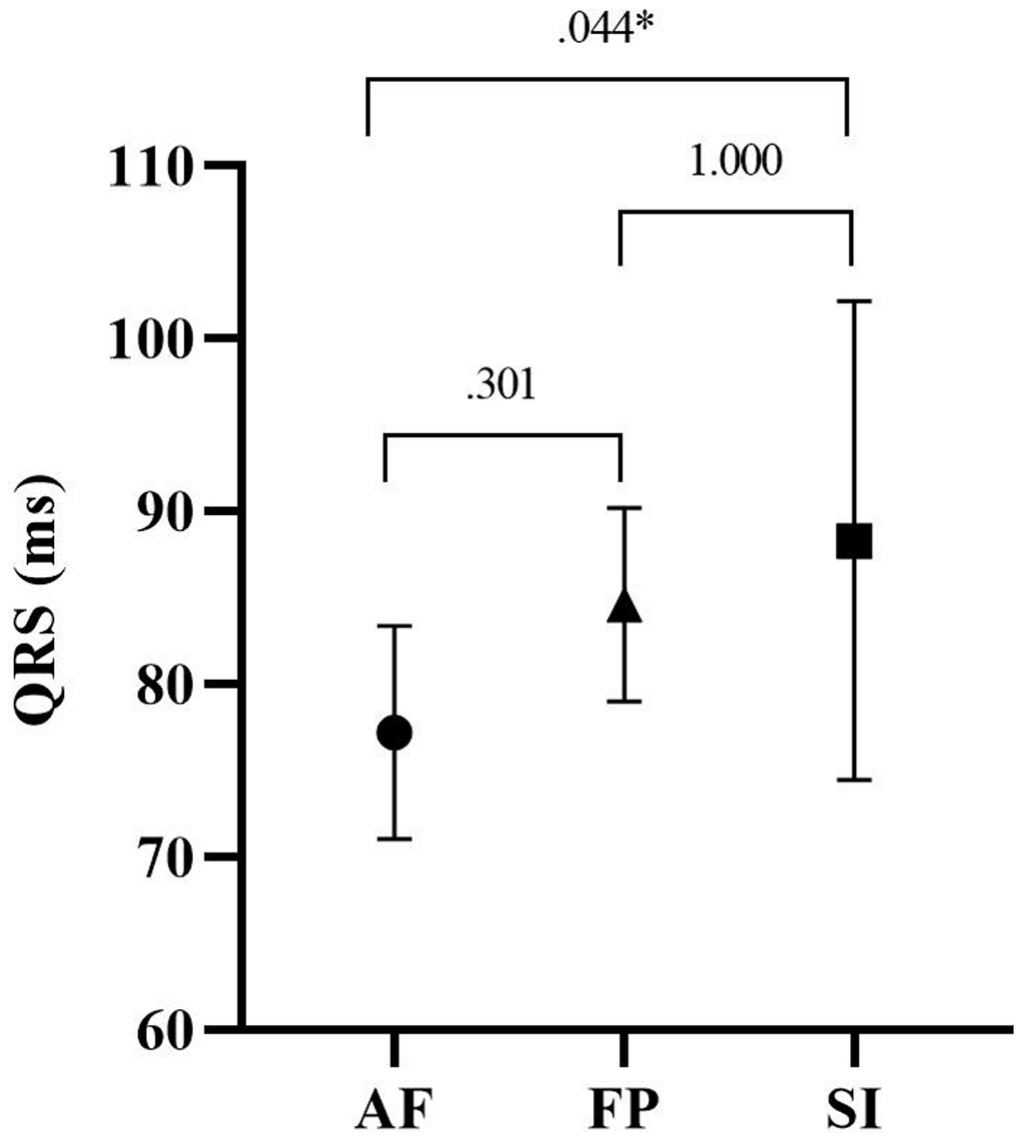
QRS durations of the study groups.

Figure 3 shows the results of the *post-hoc* test of the QRS complex durations of the participants. According to this, the QRS duration of the SI group (88.33 ± 13.87) was longer than that of the AF (77.22 ± 6.18) and HF (84.63 ± 5.60) groups. However, a significant difference was found only between AF and SI (*p* = 0.044).

## 4. Discussion

In the present study, we studied a wide range of ECG parameters in amputee football players (AF), nondisabled football players (FP) and nondisabled sedentary individuals (SI). Although most of the ECG parameters were within normal limits, QTc and P-wave length were significantly higher in AF while QRS duration was lower in AF. Although none of the players in amputee group did not show any major electrocardiographic abnormalities, this preliminary study revealed that some ECG parameters were altered in AF, which may reflect subtle physiological changes in cardiovascular system. To the best of our knowledge, this is the first study evaluating ECG findings, especially QT, QTc and Tp-e values in AF.

Contraction of the heart ventricles lasts from the beginning of the Q wave to the end of the T wave in ECG. The QRS wave represents electrical depolarization of the ventricles while T wave represents electrical repolarization of the ventricles. So, the QT interval is the sum of both the electrical depolarization and repolarization time of the ventricular muscle. Because this change may vary with heart rate, the QT interval should be corrected for heart rate (Desai et al., 2003).

It is known that some physiological changes in the body are observed during exercise (Macinnis et al., 2017). Markers of the cardiovascular system provide important insights in the analysis of physiological changes (Lavie et al., 2015; Romero et al., 2017). The ECG, which is an important method for monitoring cardiac changes in athletes, is important for interpreting structural and electrical changes in the heart that usually occur as an adaptation to regular physical activity (Maron and Pelliccia, 2006; Corrado et al., 2010). Abnormalities in ECG indicators before participation in sports or during exercise may also reflect cardiac disease causing risk of sudden cardiac death (Corrado et al., 1990; Maron, 2003; Corrado and Zorzi, 2017). The QTc values may change in both physiological and pathological conditions. Chronic exercise training in athletes has been associated with longer QT interval, whereas quitting exercise has been related to normalization of ECG with reappearance of repolarization abnormalities after beginning of exercise training (Christou et al., 2022). QT prolongation in athletes has been linked with decreased QT dispersion and thus diminished arrhythmic potential, implying the benign nature of this physiological adaptation to chronic exercise training (Stolt et al., 1999). On the contrary and pathologically, QT prolongation in individuals with congenital long QT syndrome (LQTS) is associated with increased incidence of sudden cardiac death (SCD). Although controversies still exist about the appropriate cut-off to be used to differentiate between a pathological and a physiological QTc interval, particularly in young athletes, a QTc interval > 480 ms is considered clearly abnormal even in the absence of any clinical symptoms. Subjects with a QTc ≥ 480 ms should be disqualified from competitive sports and be further examined (Corrado et al., 2010). In our study, although QTc was longer in amputee football players than in nondisabled players, QTc was within normal limits (<480 ms) and this is a reflection of benign physiological adaptation and does not carry a risk for any arrhythmic event.

Tp-e interval can be used as an index of total dispersion of ventricular repolarization (Kors et al., 2008). Prolonged Tp-e interval is associated with an increased ventricular tachyarrhythmias and cardiovascular mortality (Smetana et al., 2011). New indexes, the Tp-e/QT and Tp-e/QTc ratios, have been suggested to be more accurate measure for the dispersion of ventricular repolarization compared to Tp-e interval (Gupta et al., 2008). In our study, Tp-e, Tp-e/QT, and Tp-e/QTc were similar between groups and shows similar ventricular repolarization characteristics among groups. This again confers no risk for AF players for future cardiac arrhythmias.

The P wave on ECG is formed by electrical potentials generalized by depolarization and subsequently contraction of the right and left atriums (Israel et al., 2005). Analysis of P-wave morphology can reveal important information about atrial depolarization. Increase in P-wave duration may occur in pathological conditions such as nonhomogeneous and discontinuous atrial conduction leading to atrial enlargement and may be harbinger for atrial arrhythmias such as atrial fibrillation (Carmona Puerta et al., 2021). In our study, we found that P-wave duration is not significantly different between AF players and other nondisabled groups. This indicates there is no enlargement in atrial chambers of the heart in AF group. This was indeed was further confirmed in our previous echocardiography study in AF population (Kurtoğlu et al., 2023). However, P-wave amplitude was higher in amputee football players than in other two disabled groups though it was not a pathological elevation. This may be due to the constriction that occurs in the veins narrows the volume of space in the veins so that excess blood is pushed toward the heart, resulting somewhat an increase in cardiac output (Hecher et al., 1995).

The QRS complex on ECG occurs during depolarization of the right and left ventricles (Desai et al., 2003). Both ventricles contract rapidly to enhance cardiac output such that total ventricular contraction time or QRS duration does not exceed 100 ms. A prolongation in the QRS duration has been shown to be related a decrease in systolic functions of the left ventricle (Murkofsky et al., 1998). In our study, QRS duration was significantly lower in the amputee football players. The potential responsible mechanism for this may be due to increased peripheral vascular resistance (Li et al., 2022) resulting from amputation in amputees and increased sympathetic tone that causes cardiac tachycardia and subsequently an increase in myocardial contractile force (Lambert et al., 2014). In our study, QRS duration was similar between groups and reflects normal pump function of the heart.

In our study, the AF group was compared with healthy individuals. In a recent study, AF individuals and healthy football players and sedentary individuals were compared with echocardiography, and left ventricular end diastolic diameter and early diastole values of AFs were found to be lower than healthy football players and sedentary individuals, and left ventricular posterior wall thickness was found to be higher in AF group (Kurtoğlu et al., 2023). These results confirm that individuals with amputation have a different cardiac structure than healthy individuals. For this reason, it is thought that chronic exercises will cause different structural changes in cardiac structure in individuals with amputation compared to healthy individuals. Although some subtle structural abnormalities in echocardiography were present in AF players in our previous study, these changes were not reflected to electrocardiography. Perhaps, this may indicate that electrocardiography may not show thoroughly underlying pathophysiological processes in this population.

We should address some study limitations in the current study. Firstly, because of the homogenous study population, our results should not be generalized to female athletes or athletes practicing other sports. Secondly, this research was conducted on individuals with different amputations. There is a need for further studies examining the effects of amputation rate on cardiac structure. The third limitation of our study is that we were unable to include sedentary amputees due to difficulties in reaching this population, which may limit the generalizability of our findings. Therefore, future studies should aim to include sedentary amputees to provide a more comprehensive understanding of the topic. Nevertheless, our study provides preliminary data on a broad variety of ECG parameters in amputee football players, which can serve as a starting point for further research.

## 5. Conclusion

In conclusion, the present study showed that some ECG parameters, QTc and QRS duration, P-wave amplitude, in the amputee players differed from nondisabled subjects. A slight increase in QTc interval and a decrease in the QRS duration did not reach a pathological level and reflects a benign physiological adaptation and does not carry a risk for any arrhythmic event in the light of current data. In addition, ventricular repolarization parameters, Tp-e, Tp-e/QT, and Tp-e/QTc did not differ among groups and further confirm this finding. An increase in P-wave amplitude also reflects benign physiological adaptation. Further studies with more participants are needed to evaluate electrocardiographic parameters and set cut-off values in amputee players.

## Data availability statement

The original contributions presented in the study are included in the article/Supplementary material, further inquiries can be directed to the corresponding authors.

## Ethics statement

The necessary permissions were obtained from the Ethics Committee for Non-Interventional Research of Bandırma Onyedi Eylul University (ethical number 2022-154). The patients/participants provided their written informed consent to participate in this study.

## Author contributions

AK, ÖE, BÇ, EK, and AA: conceptualization. AK, ÖE, EK, and BÇ: methodology. AK and ÖE: formal analysis. AK, BÇ, ÖE, EK, and AA: investigation. AK, EK, ÖE, and AA: writing—original draft preparation. AK, EK, AA, ÖE, BÇ, IS, CC, DA, and IC: writing—review and editing. All authors contributed to the article and approved the submitted version.

## Conflict of interest

The authors declare that the research was conducted in the absence of any commercial or financial relationships that could be construed as a potential conflict of interest.

## Publisher’s note

All claims expressed in this article are solely those of the authors and do not necessarily represent those of their affiliated organizations, or those of the publisher, the editors and the reviewers. Any product that may be evaluated in this article, or claim that may be made by its manufacturer, is not guaranteed or endorsed by the publisher.
